# Prevalence of abnormal and borderline electrocardiogram changes in 13, 079 Chinese amateur marathon runners

**DOI:** 10.1186/s13102-021-00268-2

**Published:** 2021-04-20

**Authors:** Xu Wen, Yu-min Huang, Tong-Hui Shen, Ying-Lan Gong, Rui-qing Dong, Ling Xia, Tian-sheng Xie

**Affiliations:** 1 Department of Sport Science, College of Education, Zhejiang University, Hangzhou, China; 2 School of Sport, Exercise and Health Sciences, Loughborough University, Leicestershire, UK; 3 College of Biomedical Engineering & Instrument Science, Zhejiang University, Hangzhou, China; 4 Dushu Lake Hospital Affiliated to Soochow University, Suzhou, China; 5 Zhejiang Sino-German Institute of Life science and Healthcare, School of Biological and Chemical Engineering, Zhejiang University of Science and Technology, Hangzhou, China

**Keywords:** ECG, Marathon, Endurance exercise, Screening

## Abstract

**Background:**

The 12-lead electrocardiogram (ECG) has been adopted as an important component of preparticipation cardiovascular screening. However, there are still controversies in the screening and few studies with a large sample size have reported the results of ECGs of marathon runners. Therefore, the purpose of this study was to assess the prevalence of normal, borderline, and abnormal ECG changes in marathon runners.

**Methods:**

The 12-lead ECG data of 13,079 amateur marathon runners between the ages of 18 and 35 years were included for analysis. The prevalence of ECG abnormalities among different gender groups was compared with chi-square tests.

**Results:**

In terms of training-related changes, sinus bradycardia, sinus arrhythmia, and left ventricular high voltage were found in approximately 15, 5, and 3.28% of the participants, respectively. The incidence of right axis deviation in the marathon runners was 1.78%, which was slightly higher than the incidence of left axis deviation (0.88%). No more than 0.1% of the amateur marathon runners exhibited ST-segment depression, T wave inversion (TWI), premature ventricular contraction, pathologic Q waves, and prolonged QT interval.

**Conclusions:**

Training-related ECG changes, including sinus bradycardia, sinus arrhythmia, and left ventricular high voltage, were common in amateur marathon runners. Most abnormal ECG changes, including ST-segment depression, TWI, premature ventricular contraction, pathologic Q waves, and prolonged QT interval, were infrequently found in amateur marathon runners. The data also suggested Chinese amateur marathon runners may have a relatively lower prevalence of ECG abnormalities than black and white runners.

## Background

Marathon is generally becoming one of the most popular vigorous endurance training in the world. However, prolonged vigorous endurance training may lead to adverse functional and morphological cardiac adaptations, especially for amateur runners [1]. The incidence of sudden death in marathon runners ranges from 0.54 to 2.1/100,000, with cardiovascular diseases (CVDs) considered as one of the major triggers during long-distance competitions [2–4]. Although the risk of sudden cardiac death (SCD) during a sporting event is low, owing to media coverage, people may overestimate the risk of participating in a marathon and reduce their participation in sports.

Preparticipation screening examination might be specifically important to decrease the risk of sudden death during the competitions. The annual incidence of SCD in athletes decreased by 89% in Italy after a nationwide systematic preparticipation screening examination was introduced [5]. Superiority in simple operation, excellent quality and reasonable budget, the 12-lead electrocardiogram (ECG) was recommended as an objective test to increase sensitivity and specificity for preparticipation cardiovascular screening. Except personal history, family history, and physical examination, ECG provided important diagnostic and prognostic information for a variety of CVDs. While, there were still several controversies in preparticipation cardiovascular screening. Opponents criticized the ECG screening for its limitations including high costs, high false-positive rate, potential false negatives, and lack of large sample study [6]. Therefore, research data is still needed to assess the utility of the screening program and to optimize the ECG screening criteria.

Over the past decades, ECG interpretation standards for athletes were established and improved to identify potentially life-threatening pathologic cardiovascular disorders associated with SCD in athletes [7]. The ECG abnormalities were not recognized features of athletic training and always require further assessment to exclude the presence of intrinsic cardiac disease. Numerous studies have reported the prevalence of ECG abnormalities in athletes of different sports [8–10]. Several studies have likewise reported ECG abnormalities in marathon runners. Minns et al. reported the prevalence of ECG abnormalities in 87 volunteer runners before and after a half marathon [11]. Herm et al. presented the ECG results of 108 athletes during a marathon race with portable ECG recorders [12]. Although millions of runners participate in thousands of marathons every year, few studies with a large sample size have reported the results of ECGs of marathon runners.

Racial and gender differences in cardiovascular risk exist among athletes. The incidence of SCD is higher in black Americans compared with white Americans [13]. Moreover, abnormal T wave inversion (TWI) is more frequent in black (12%) than in white competitive athletes (< 4%) [14, 15]. Thus, determining the prevalence of ECG abnormalities in other ethnicities would be interesting. However, ECG findings in Hispanic and Asian athletes as well as in those with mixed ethnicities were not well reported [16]. Moreover, it might be beneficial to allocating medical resources reasonably, providing medical services efficiently via determining the prevalence of borderline and abnormal ECG changes. Therefore, this study aims to describe the prevalence of normal, borderline, and abnormal ECG changes in young Chinese marathon runners, which may serve as a reference for preparticipation screening for marathon events.

## Methods

### Participants

The Hangzhou Marathon first held in 1987 is one of the earliest marathon events for amateur enthusiasts in China. There are approximately 20,000 runners enrolling into the marathon’s competitions every year. Except for a very small number of professional elite athletes participating in the Hangzhou Marathon, most of the participants are ordinary amateur runners to improve health situations, gaining happiness or challenging themselves. The participants were 18,826 contestants from 2015 and 2016 Hangzhou Marathon (half and full categories) competitions. Before the marathon, the amateur runners were required to submit their physical examination report (a 12-lead resting ECG was strongly recommended) through the organiser website for preparticipation screening. The inclusion criteria for the participants in the current study included the following: (1) age was between 18 and 35 years old; (2) submitting 12-lead ECG report (In China, patients would get an ECG report sheet with 12-lead electrocardiogram after taking an ECG exam in their local hospitals); (3) the ECG test was conducted in a qualified county-level hospital or higher within 6 months; (4) accessing to personal information (e.g. gender, age, height, and weight); and (5) only the 2016 data were included if participants both enrolled into the 2015 and 2016 events. All participants read and signed an informed consent form and this study was approved by the Ethics Committee of the First Affiliated Hospital of the Medical School of Zhejiang University.

### Procedures

The data analysis of this study was based on the ECGs, submitted by the marathon runners. The ECGs were interpreted by a group of clinical doctors and verified by reviewers with the international recommendation for ECG interpretation in athletes released in 2017 [17]. In cases with inconsistent diagnoses, a third reviewer was responsible for interpreting and making decisions. Personal information, including gender, age, height, and weight, was collected from the physical examination reports.

### Data analysis

The prevalence of normal, borderline and abnormal ECG changes was presented, and chi-square tests were applied to compare prevalence rates between the male and female runners. Using the median running time of the full or half marathons as the cutoff point, the participants were divided into good and poor performance groups. Logistic regression models were utilized to determine the association between certain ECG abnormalities and sports performance in marathon. Sports performance and ECG abnormality were considered as the independent factors. Odds ratios of having ECG findings were determined in marathon runners with good performance as compared with their counterparts with poor performance, in which *p* values less than 0.05. All analyses were done with SPSS 20.0 (SPSS Inc.).

## Results

A total of 13,079 adults, including 4896 full marathon runners and 8183 half marathon runners, meet the inclusion criteria in this study. 64.6% of the participants were young adults no older than 30 years (Table 1). More than three-quarters of the participants were men. Although the study population was composed of marathon runners, more than 20% of the male runners were overweight. Besides, most runners are still entry-level marathon enthusiasts, who had not experienced long-term intensive training. About 75% of runners took more than 4 h to complete the full marathon and nearly 80% of runners need over 2 h to finish the half marathon.

**Table 1:** Characteristics of the study population

	Men	Women	Total
n	9897 (75.7%)	3182 (24.3%)	13,079 (100%)
Full marathon	4102 (83.8%)	794 (16.2%)	4896 (100%)
Half marathon	5795 (70.8%)	2388 (29.2%)	8183 (100%)
Age, years			
Mean ± SD	28.6 ± 4.2	27.9 ± 4.1	28.5 ± 4.2
< 30 years	6220 (62.8%)	2232 (70.1%)	8452 (64.6%)
30–35 years	3677 (37.2%)	950 (29.9%)	4627 (35.4%)
Height, cm	173.6 ± 5.2	162.5 ± 4.8	170.6 ± 6.7
Weight, kg	67.9 ± 8.9	54.8 ± 8.6	65.0 ± 10.0
BMI, kg/m^2^	22.4 ± 2.2	20.3 ± 2.2	22.2 ± 2.2
Overweight, n(%)	1853 (20.3%)	74 (2.5%)	1927 (16.0%)
Obesity, n(%)	94 (1.0%)	25 (0.7%)	114 (0.9%)
Finishing time			
Full marathon, Mean (SD)	4h22min (42 min)	4h47min (39 min)	4h26min (42 min)
< 3 h	25 (0.6%)	2 (0.3%)	27 (0.6%)
3–4 h	1120 (27.3%)	67 (8.4%)	1187 (24.2%)
4–5 h	1955 (47.7%)	361 (45.5%)	2316 (47.3%)
> 5 h	1002 (24.4%)	364 (45.8%)	1366 (27.9%)
Half marathon, Mean (SD)	2h10min(20 min)	2h22min (17 min)	2h14min (20 min)
< 2 h	1604 (27.7%)	152 (6.4%)	1756 (21.5%)
2–3 h	4143 (71.5%)	2187 (91.6%)	6330 (77.4%)
> 3 h	48 (0.7%)	49 (2.0%)	97 (1.2%)

Note: overweight: BMI ≥ 24 kg/m^2^ and < 28 kg/m^2^; obesity: BMI ≥ 28 kg/m^2^

According to the 2017 international recommendation for ECG interpretation, the prevalence of training-related changes was shown in Table 2. Sinus bradycardia and sinus arrhythmia were found in approximately 14.67 and 8.09% of the participants, respectively. The prevalence of left ventricular high voltage was 3.12%, and the male runners exhibited a significantly higher incidence (4.04%) compared with the female runners (0.25%). 1.25% of runners were found to have early repolarisation. No more than 1% of the runners demonstrated first-degree atrioventricular block (AVB), Mobitz type 1 (Wenckebach) second-degree AVB, ectopic atrial or junctional rhythm, and incomplete right bundle branch block (IRBBB). It is noteworthy that, the prevalence of sinus bradycardia and sinus arrhythmia in normal weight runners is 15.8 and 6.0% respectively, which are significantly higher than the prevalence in overweight runners (14.3 and 4.0%). The results of logistic regression illustrated that the good-performance group presented higher rates for sinus bradycardia, left ventricular high voltage, right ventricular high voltage, and AVB after the age, gender, and weight status were adjusted in the models (Table 3).

**Table 2:** Prevalence of training related ECG changes in the male and female marathon runners

	Men (*n* = 9897)	Women (*n* = 3182)	Total (*n* = 13,079)	P^*^
	n %	n %	n %	
Sinus bradycardia	1544 15.60%	375 11.79%	1919 14.67%	< 0.01
Sinus arrhythmia	790 7.98%	268 8.42%	1058 8.09%	> 0.05
Left ventricular high voltage	400 4.04%	8 0.25%	408 3.12%	< 0.01
Right ventricular high voltage	9 0.09%	0 0.00%	9 0.07%	> 0.05
Early Repolarization/ ST segment elevation	130 1.31%	33 1.04%	163 1.25%	> 0.05
1° AVB	39 0.39%	4 0.13%	43 0.33%	< 0.01
Mobitz type 1 (Wenckebach) 2° AVB	1 0.01%	0.00%	1 0.01%	
Ectopic atrial or junctional rhythm	5 0.05%	1 0.03%	6 0.05%	> 0.05
Incomplete RBBB	118 1.19%	9 0.28%	127 0.97%	< 0.01
Any of the above findings	2071 20.93%	546 17.16%	20.01%	< 0.01

Some participants had to have more than one ECG abnormality

Note: *: *p* value of chi-square test between men and women

*AVB* atrioventricular block, *RBBB* right bundle branch block

**Table 3:** Odds ratios of having normal or abnormal ECG findings in marathon runners with good performance using the runners with poor performance as reference

	OR	95% confidence interval
Normal ECG	1.346	1.261–1.438
Sinus bradycardia	1.518	1.409–1.636
Sinus arrhythmia	1.010	0.899–1.135
left ventricular high voltage	1.436	1.233–1.672
1° AVB	1.702	1.034–2.804
Incomplete RBBB	0.803	0.606–1.065
right axis deviation	0.917	0.717–1.174
left axis deviation	0.869	0.611–1.236

Note: Age, gender, weight status were adjusted in the logistic regression model

*AVB* Atrioventricular block, *RBBB* Right bundle branch block

The prevalence of borderline ECG findings is shown in Table 4. The incidence of right axis deviation in the marathon runners was 1.78%, which was slightly higher than the incidence of left axis deviation (0.88%).0.70, and 0.54% of runners were found to have left and right atrial enlargement respectively. A total of 18 men and only 1 woman were found to have the complete right bundle branch block (CRBBB).

**Table 4:** Prevalence of borderline ECG findings in the male and female marathon runners

	Men (*n* = 9897)	Women(*n* = 3182)	Total(*n* = 13,079)	P^*^
	n %	n %	n %	
Right axis deviation	176 1.78%	28 0.88%	204 1.56%	< 0.01
Left axis deviation	98 0.99%	9 0.28%	107 0.82%	< 0.01
Left atrial enlargement	70 0.71%	22 0.69%	92 0.70%	> 0.05
Right atrial enlargement	55 0.56%	16 0.50%	70 0.54%	> 0.05
Complete RBBB	18 0.18%	1 0.03%	19 0.15%	< 0.05
Any of the above findings	386 3.90%	66 2.07%	452 3.46%	< 0.01

Note: *: *p* value of chi-square test between men and women

*RBBB* right bundle branch block

The prevalence of abnormal ECG findings was very low, and none was greater than 1‰ (Table 5). Among the amateur runners, 0.03% (2 men and 2 women) were found to have TWI. It was noteworthy that 6 (0.06%) male and 6 (0.19%) female runners were found to have ST-segment depression. Also, several runners were found to have a premature ventricular contraction, pathologic Q waves, atrial fibrillation, and prolonged QT interval.

**Table 5:** Prevalence of abnormal ECG findings in the male and female marathon runners

	Men (*n* = 9897)	Women (*n* = 3182)	Total (*n* = 13,079)	P^*^
	n %	n %	n %	
T wave inversion	2 0.02%	2 0.06%	4 0.03%	> 0.05
ST segment depression	6 0.06%	6 0.19%	12 0.09%	< 0.01
Pathologic Q waves	2 0.02%		2 0.02%	
Premature ventricular contraction	4 0.04%	1 0.03%	5 0.04%	> 0.05
Atrial fibrillation	1 0.01%		1 0.01%	
Prolonged QT interval	1 0.01%		1 0.01%	
Any of the above findings	15 0.15%	7 0.22%	22 0.17%	> 0.05

Note: *: *p* value of chi-square test between men and women

## Discussion

Currently, the prevalence of training-related, borderline, and abnormal ECG changes in a large sample of amateur marathon runners were reported to serve as a reference for SCD prevention during marathons. It was noteworthy that the prevalence of some ECG changes in the current study were extremely low. Only 0.97 and 0.15% of the runners were found to have IRBBB and CRBBB, respectively, which was lower than the prevalence reported in previous studies (IRBBB: 7–20%, CRBBB: 2–3%) [18–20]. The prevalence of AVB in the athletes in this study (0.34%) was also significantly lower than that reported in previous studies (approximately 4.5–7.5%) and similar to that in sedentary healthy adults in other studies [10, 14, 21, 22]. Low prevalence was also found in ST-segment depression, TWI, and pathologic Q waves. TWI was found in 22.9% of 329 adolescent black athletes and 4.5% of 903 adolescent white athletes [23]. Another study using 1000 athletes showed that the prevalence of TWI was 4% [10]. However, in the current study, no more than 0.1% of the amateur marathon runners exhibited ST-segment depression, TWI, premature ventricular contraction, pathologic Q waves, and prolonged QT interval.

The large difference in prevalence rates was attributed to multiple reasons, such as participants’ training levels. Lots of ECG changes including AVB, RBBB, and ST-segment elevation are associated with long-term endurance training [24, 25] and could be influenced by the number of years of training in a respective sport [20]. In the current study, more than 75% of participants took more than 4 h to complete full a marathon and nearly 80% of runners need more than 2 h to finish the half marathon. Therefore, most participants in this study were physical active adults and different from professional athletes who receive long-term intensive training. Due to the U-shaped relationship between physical activity and cardiac abnormality morbidity [26], the physical active participants may have a significant lower prevalence of abnormal ECG findings than sedentary Chinese adults and professional athletes.

The data of this study confirmed that the prevalence of borderline changes and abnormal changes in amateur marathon runners are lower than the prevalence in the ordinary Chinese population [27, 28]. For instance, it was found that the prevalence of CRBBB (men: 0.18%, women: 0.03%) and atrial fibrillation (men: 0.01%, women: 0%) is significantly lower than the prevalence in ordinary Chinese (CRBBB: men: 0.51%, women: 0.35%; atrial fibrillation: men: 0.14%, women: 0.08%) reported by Yu and her colleagues [28]. The data also confirmed that the prevalence of training related ECG changes including sinus bradycardia (men: 15.60%, women: 11.79%) and sinus arrhythmia (men: 7.98%, women: 8.42%) are much more common in amateur marathon runners as compared with ordinary Chinese with similar age (sinus bradycardia: men: 3.99%, women: 1.62%; atrial fibrillation: men: 0.94%, women: 1.78%).

Numerous studies indicated that ECG abnormalities vary across ethnicity and these variations may have implications for further diagnostic testing [7, 29]. Electrocardiographic repolarisation changes and echocardiographic left ventricular hypertrophy have been demonstrated to be more common in black athletes [15, 30]. However, most of these studies were conducted among the black and white athletes, lacking enough ECG data from Chinese athletes. The prevalence of TWI reported by Feng et al. was 1.7% in young Chinese athletes and none of the college students was found to have TWI [31]. While 4.5% of West-Asian young athletes and 15.9% of black athletes were found to have TWI [32]. The prevalence of ECG abnormalities was found relatively lower among the Chinese population when compared with South Asians groups [33]. Moreover, pathologic Q waves may also be infrequently in Chinese. Ng et al. reported that only 20 out of 18,476 young male military conscripts had abnormal Q waves [33]. Another study demonstrated that the prevalence of pathologic Q waves was 1.8% in ordinary Chinese (35–54 years old) which was also significantly lower than the data in the U.S. [27]. These research shreds of evidence suggested Chinese populations may have a relatively low prevalence of ECG abnormalities.

Also, the possibility of selection bias may also be an important reason for the low prevalence of ECG abnormalities found in this study. The data of this study came from the self-submitted ECG report by the marathon runners. Therefore, the prevalence of ECG abnormalities could be underestimated, as some marathon runners maybe not willing to submit their ECG report if they had serious cardiac problems.

It is noteworthy that there were no obvious changes regarding prevalence of most normal and borderline ECG in the current studies. Left and right axes deviation is classified by the new criteria as a borderline training-related ECG change. Gati et al. reported that the prevalence of left and right axes deviation in 2533 athletes was 1.46 and 1.11%, respectively [34]. In the current study, 1.56 and 0.82% of the marathon runners were found to have right and left axes deviation respectively, which is similar to the results reported by Gati et al. Furthermore, the data suggested that no significant differences existed in the incidence of left and right axes deviation between amateur marathon runners and sedentary healthy adults [21, 22, 35]. It confirmed that long-term marathon training caused physiological changes in cardiac function, reflecting in ECG training-related changes. Sinus bradycardia, sinus arrhythmia, and training-related ECG changes were associated with the increased vagal tone, being common in long-distance runners [36]. The prevalence of sinus bradycardia in sedentary healthy Chinese adults is 5.99–10.51% [22, 37], which was lower than that in the marathon runners (14.67%). The prevalence of sinus arrhythmia in the participants was 8.09%, which is similar to that in sedentary healthy adults (5.38–8.14%) [22, 37].

This study has several limitations. Firstly, the process of data collection was not standardized. Although Chinese hospitals implement very similar operation standard of ECG examination [38], the ECG examination was conducted in different hospitals and the reports were submitted by the participants. Moreover, due to the self-submitted ECG data, the prevalence of ECG abnormalities could be underestimated. Thirdly, all the participants were Chinese in this study. As ethnicity is an important determinant of cardiac adaptation to exercise [23, 39], the results may not be generalised to other ethnicities. Fourthly, further cardiovascular examinations were not conducted in participants with positive ECG results, so the true disease rate is unclear. Moreover, the medium marathon completion time was applied as the cut-off point to divide the runners into good and poor groups because the marathon completion time in the participants was not normally distributed and most of the participants are amateurs with low performance. In addition, another limitation is the lack of a control group in the study design.

This study had several notable strengths. The size and demographic diversity of sample are significant. Despite these limitations, our study provided the new perspective in understanding the cardiac status of amateur marathon runners, offering the platform for future investigation in exploring the influence of prolonged marathon training on the heart, and the prevention of SCD during marathon events.

## Conclusion

Training-related ECG changes, including sinus bradycardia, sinus arrhythmia, and left ventricular high voltage, were common in amateur marathon runners. Besides, left and right axes deviation, as well as atrial enlargement, were common borderline ECG changes. Most abnormal ECG changes, including ST-segment depression, TWI, premature ventricular contraction, pathologic Q waves and prolonged QT interval, were infrequently found in amateur marathon runners. The data also suggested Chinese amateur marathon runners may have a relatively lower prevalence of ECG abnormalities than black and white runners.

## Data Availability

The datasets used and/or analyzed during the current study are available from the corresponding author on reasonable request.

## Abbreviations

ECG: Electrocardiogram
TWI: T wave inversion
SCD: sudden cardiac death
AVB: Atrioventricular block
IRBBB: Incomplete right bundle branch block
CRBBB: Complete right bundle branch block

## References

[1] Predel HG (2014). Marathon run: cardiovascular adaptation and cardiovascular risk. Eur Heart J.

[2] Kim JH, Malhotra R, Chiampas G, d'Hemecourt P, Troyanos C, Cianca J, Smith RN, Wang TJ, Roberts WO, Thompson PD, Baggish AL (2012). Cardiac arrest during long-distance running races. N Engl J Med.

[3] Webner D, DuPrey KM, Drezner JA, Cronholm P, Roberts WO (2012). Sudden cardiac arrest and death in United States marathons. Med Sci Sports Exerc.

[4] Dayer MJ, Green I (2019). Mortality during marathons: a narrative review of the literature. BMJ Open Sport Exerc Med.

[5] Corrado D, Basso C, Pavei A, Michieli P, Schiavon M, Thiene G (2006). Trends in sudden cardiovascular death in young competitive athletes after implementation of a Preparticipation screening program. JAMA..

[6] Petek BJ, Baggish AL (2020). Current controversies in pre-participation cardiovascular screening for young competitive athletes. Expert Rev Cardiovasc Ther.

[7] Sharma S, Drezner JA, Baggish A, Papadakis M, Wilson MG, Prutkin JM, la Gerche A, Ackerman MJ, Borjesson M, Salerno JC, Asif IM, Owens DS, Chung EH, Emery MS, Froelicher VF, Heidbuchel H, Adamuz C, Asplund CA, Cohen G, Harmon KG, Marek JC, Molossi S, Niebauer J, Pelto HF, Perez MV, Riding NR, Saarel T, Schmied CM, Shipon DM, Stein R, Vetter VL, Pelliccia A, Corrado D (2018). International recommendations for electrocardiographic interpretation in athletes. Eur Heart J.

[8] Maron BJ, Bodison SA, Wesley YE, Tucker E, Green KJ (1987). Results of screening a large group of intercollegiate competitive athletes for cardiovascular disease. J Am Coll Cardiol.

[9] Molinari G, Brunetti ND, Biasco L, Squarcia S, Cristoforetti Y, Bennicelli R, del Vecchio C, Viacava C, Giustetto C, Gaita F (2017). Electrocardiograms of children and adolescents practicing non-competitive sports: Normal limits and abnormal findings in a large European cohort evaluated by Telecardiology. Sports Med.

[10] Sharma S, Whyte G, Elliott P, Padula M, Kaushal R, Mahon N, McKenna WJ (1999). Electrocardiographic changes in 1000 highly trained junior elite athletes. Br J Sports Med.

[11] Minns AB, Mcfarland C, Strachan M, Austin W, Castillo E, Ben-Yehuda O (2011). Electrocardiogram and echocardiogram findings in runners completing a half marathon. Am J Emerg Med.

[12] Herm J, Topper A, Wutzler A, Kunze C, Krull M, Brechtel L (2017). Frequency of exercise-induced ST-T-segment deviations and cardiac arrhythmias in recreational endurance athletes during a marathon race: results of the prospective observational Berlin beat of running study. BMJ Open.

[13] Harmon KG, Drezner JA, Wilson MG, Sharma S (2014). Incidence of sudden cardiac death in athletes: a state-of-the-art review. Br J Sports Med.

[14] Papadakis M, Basavarajaiah S, Rawlins J, Edwards C, Makan J, Firoozi S, Carby L, Sharma S (2009). Prevalence and significance of T-wave inversions in predominantly Caucasian adolescent athletes. Eur Heart J.

[15] Papadakis M, Carre F, Kervio G, Rawlins J, Panoulas VF, Chandra N, Basavarajaiah S, Carby L, Fonseca T, Sharma S (2011). The prevalence, distribution, and clinical outcomes of electrocardiographic repolarization patterns in male athletes of African/afro-Caribbean origin. Eur Heart J.

[16] Panhuyzen-Goedkoop NM, Jorstad HT, Smeets JLRM (2018). A new consensus document on electrocardiographic interpretation in athletes: does it help to prevent sudden cardiac death in athletes?. Neth Hear J.

[17] Drezner JA, Sharma S, Baggish A, Papadakis M, Wilson MG, Prutkin JM, Gerche AL, Ackerman MJ, Borjesson M, Salerno JC, Asif IM, Owens DS, Chung EH, Emery MS, Froelicher VF, Heidbuchel H, Adamuz C, Asplund CA, Cohen G, Harmon KG, Marek JC, Molossi S, Niebauer J, Pelto HF, Perez MV, Riding NR, Saarel T, Schmied CM, Shipon DM, Stein R, Vetter VL, Pelliccia A, Corrado D (2017). International criteria for electrocardiographic interpretation in athletes: consensus statement. Br J Sports Med.

[18] Kim JH, Noseworthy PA, Mccarty D, Yared K, Weiner R, Wang F (2011). Significance of electrocardiographic right bundle branch block in trained athletes. Am J Cardiol.

[19] Pelliccia A, Maron BJ, Culasso F, Paolo FMD, Spataro A, Biffi A (2000). Clinical significance of abnormal electrocardiographic patterns in trained athletes. Circulation..

[20] Langdeau JB, Blier L, Turcotte H, O'Hara G, Boulet LP (2001). Electrocardiographic findings in athletes: the prevalence of left ventricular hypertrophy and conduction defects. Can J Cardiol.

[21] Hingorani P, Karnad DR, Natekar M, Kothari S, Narula D (2014). Baseline and new-onset morphologic ECG abnormalities in healthy volunteers in phase I studies receiving placebo: changes over a 6-week follow-up period. J Clin Pharmacol.

[22] Shen SY, Wu Q (2009). Analysis of electrocardiogram in 5664 health Chinese adults. J Pract Electrocardiol.

[23] Sheikh N, Papadakis M, Carre F, Kervio G, Panoulas VF, Ghani S, Zaidi A, Gati S, Rawlins J, Wilson MG, Sharma S (2013). Cardiac adaptation to exercise in adolescent athletes of African ethnicity: an emergent elite athletic population. Br J Sports Med.

[24] Doutreleau S, Pistea C, Lonsdorfer E, Charloux A (2013). Exercise-induced second-degree atrioventricular block in endurance athletes. Med Sci Sports Exerc.

[25] Pappas LK, Efremidis M, Sideris A, Letsas KP, Kounas SP, Kardaras F (2006). Exercise-induced second-degree atrioventricular block. Int J Cardiol.

[26] Merghani A, Malhotra A, Sharma S (2015). The U-shaped relationship between exercise and cardiac morbidity. Trends Cardiovasc Med.

[27] Rao X, Wu X, Folsom AR, Liu X, Zhong H, Williams OD, Stamler J (2000). Comparison of electrocardiographic findings between northern and southern Chinese population samples. Int J Epidemiol.

[28] Yu L, Ye X, Yang Z, Xiao J, Zhang B. Prevalences and Associated Factors of Electrocardiographic Abnormalities in Chinese Adults: A Cross-sectional Study. BMC Cardiovascular Disorders**.** 2020;20:414. 10.1186/s12872-020-01698-5 PMC7488680 32917144

[29] Hebert K, Lopez B, Dias A, Steen DL, Colombo RA, Franco E, Neistein S, Arcement LM (2010). Prevalence of electrocardiographic abnormalities in a systolic heart failure disease management population by race, ethnicity, and sex. Congest Heart Fail.

[30] Chandra N, Papadakis M, Sharma S (2012). Cardiac adaptation in athletes of black ethnicity: differentiating pathology from physiology. Heart..

[31] Feng L, Ran Y, Li N, Zhang JQ, Ma Y, Ma CS (2015). Abnormal electrocardiographic patterns in trained athletes screened by European Society of Cardiology recommendations. Chin J Cardiac Pacing Electrophysiol.

[32] Wilson MG, Chatard JC, Carre F, Hamilton B, Whyte GP, Sharma S, Chalabi H (2012). Prevalence of electrocardiographic abnormalities in west-Asian and African male athletes. Br J Sports Med.

[33] Ng CT, Ong HY, Cheok C, Chua TS, Ching CK (2012). Prevalence of electrocardiographic abnormalities in an unselected young male multi-ethnic south-east Asian population undergoing pre-participation cardiovascular screening: results of the Singapore armed forces electrocardiogram and echocardiogram screening protocol. Europace..

[34] Gati S, Sheikh N, Ghani S, Zaidi A, Wilson M, Raju H, Cox A, Reed M, Papadakis M, Sharma S (2013). Should axis deviation or atrial enlargement be categorised as abnormal in young athletes? The athlete's electrocardiogram: time for re-appraisal of markers of pathology. Eur Heart J.

[35] Ostrander LD (1971). Left axis deviation: prevalence, associated conditions, and prognosis. An epidemiologic study. Ann Intern Med.

[36] Corrado D, Pelliccia A (2011). Recommendations for interpretation of 12-lead electrocardiogram in the athlete. Eur Heart J.

[37] Cui T, Zhao M-X, Wu Q, Hu J (2013). Analysis of electrocardiogram of health physical examination 18636 cases. China Heart J.

[38] Expert Group of “Guidelines for the operation of routine ECG examination” (2019). Guidelines for the operation of routine ECG examination (simplified version). J Pract Electrocardiol.

[39] Sandeep B, Araceli B, Gregory W, Mathew W, Lorna C, Ajay S (2008). Ethnic differences in left ventricular remodeling in highly-trained athletes - relevance to differentiating physiologic left ventricular hypertrophy from hypertrophic cardiomyopathy. J Am Coll Cardiol.

